# Comparative analysis of magnetized partially ionized copper, copper oxide–water and kerosene oil nanofluid flow with Cattaneo–Christov heat flux

**DOI:** 10.1038/s41598-020-74865-5

**Published:** 2020-11-09

**Authors:** Nomana Abid, Muhammad Ramzan, Jae Dong Chung, Seifedine Kadry, Yu-Ming Chu

**Affiliations:** 1 Department of Computer Science, Bahria University, Islamabad Campus, Islamabad, 44000 Pakistan; 2 Department of Mechanical Engineering, Sejong University, Seoul, 143-747 Korea; 3 Department of Mathematics and Computer Science, Faculty of Science, Beirut Arab University, Beirut, 115020 Lebanon; 4 Department of Mathematics, Huzhou University, Huzhou, 313000 People’s Republic of China; 5 Hunan Provincial Key Laboratory of Mathematical Modeling and Analysis in Engineering, Changsha University of Science and Technology, Changsha, 410114 People’s Republic of China

**Keywords:** Mechanical engineering, Mathematics and computing

## Abstract

This comparative analysis studies the impact of two different nanoparticles Copper and Copper Oxide in two different partially ionized magnetofluid (water and kerosene oil mixed with Copper/Copper Oxide) flows over a linearly stretching surface. The impacts of electrons and ions collisions in the presence of the Cattaneo-Christov heat transfer model are also investigated. The effects of prominent parameters on velocity and temperature fields are depicted through graphical illustrations. A similarity transformation procedure is applied to transform the nonlinear partial differential equations to the ordinary one. Our numerical methodology is based upon the Finite difference method that is the default method in the bvp4c built-in function of the MATLAB scheme. Nusselt number and Skin drag coefficient are computed numerically and presented in tabular form for both types of nanofluids over a linear stretched surface. Our results demonstrate that the effects of CuO are dominant in comparison to the Cu on fluid velocity. The fluid temperature is more prominent in the case of Cu-water nanofluid when we increase nanoparticles concentration.

## Introduction

Having high thermal conductivity as compared to liquids, solids are used as nanoparticles to enhance the thermal properties of the base fluids. Nanoparticles with size less than a hundred nanometers in the base fluid are the most conversed topic of today’s technological and engineering fields. Copper has been an important solid material to man since ancient times. Copper (Cu) is the oldest metal with ductile nature and has high thermal and electrical properties in comparison to other metals. At room temperature, the thermal conductivity of pure copper is 401 W/m. K, which shows that a one-meter copper sheet or wall can conduct heat at a rate of 401 W/m^2^. Copper-oxide (CuO) is produced when the copper reacts with the oxygen. The metal oxides used as catalysts in photonic and electronic devices are significant technology materials. Cu has a pinkish-orange color whereas CuO arises as a black-brownish powder. Researchers have paid more attention to the study of Cu and Cu-based nanoparticles due to their innovative technological applications. CuO nanoparticles are used in a wide range of applications i.e., batteries, catalysis, magnetic storage media, gas sensors, semiconductors, and solar energy transformer. For diverse applications, several methods have been developed to produce Cu and CuO nanoparticles^1–6^. Many researchers worked on the thermal conductivity of CuO nanoparticles for heat transfer applications. Lee et al*.*^7^ dispersed CuO nanoparticles of the size 30–80 nm in the base fluids and discovered that CuO nanofluids exhibited higher thermal conductivity in comparison to the model of Hamilton and Crosser^8^, in which the size effect of nanoparticles was neglected. Bachok et al*.*^9^ studied the stagnation-point flow within the copper–water nanofluid past a stretching sheet. Hassnain et al*.*^10^ analyzed the comparative study of Cu/Ag-water and Cu/Ag-kerosene oil nanofluids over a stretching surface. They found that Cu/Ag-kerosene oil nanofluid has a high rate of heat transfer and skin friction as compared to Cu/Ag-water nanofluid. Hayat et al*.*^11^ investigated the rate of heat transfer in CuO-Ag water-based hybrid nanofluid past a linearly stretching surface. Some more nanofluid flows highlighting Cu-CuO amalgamation with base fluids may be found in^12–16^.

The process of heat transport is basically the transmission of heat from the surface with high temperatures to the surface with low temperatures. Attention is paid to predicting the behavior of heat transport in numerous situations, owing to its significance in innumerable engineering applications, for example in the bio-medical sector for magnetic drug targeting, nuclear reactor cooling, and energy production. The law of heat conduction was first proposed by Fourier^17^ in 1822. This law provides a basic understanding of the heat transmission phenomenon and became the basis for learning on heat conduction in the next two centuries. However, the deficiency of this law was that during the heat transmission, any small disturbance is sensed immediately by the whole system which contradicts the causality principle. To tackle this deficiency Cattaneo^18^ added a thermal time relaxation parameter for a finite speed of heat transmission through any medium but it was difficult to produce a temperature equation from this law. Afterward, Christov^19^ modified Cattaneo’s law by using upper-convected Oldroyd’s^20^ derivative to yield a single equation for the temperature field. This law was titled a Cattaneo-Christov (CC) heat flux model. Zampoli and Tibullo^21^ examined the uniqueness of the CC heat flux model. Sha et al*.*^22^ investigated the Hall effect on 3D couple stress nanofluid flow with CC heat flux model past an exponentially stretching sheet. Shah et al*.*^23^ discussed the micropolar Casson ferrofluid flow with an effective thermal conductivity model and CC heat flux model over a stretching surface. The 3D MHD Darcy-Forchheimer nanofluid through an exponentially stretching sheet with CC heat flux model and zero mass flux conditions of nanoparticles is scrutinized by Ahmed et al*.*^24^. Rasool et al*.*^25^ analyzed the CC theory of mass and heat flux within Darcy-Forchheimer porous media through a non-linear stretching surface. Summayya et al*.*^26^ proposed the model of time-independent Williamson nanofluid with CC heat flux over a stretched heated surface. Ramzan et al.^27^ examined the Maxwell fluid three-dimensional flow in the existence of the CC heat flux model with the activation energy and homogeneous-heterogeneous reactions. It is observed that the present literature does not contain partially ionized nano liquid with CC heat flux over a stretching sheet.

The partially ionized fluid experiences more than one type of force when the magnetic field is applied to it. Due to the magnetic field, these forces contain magnetic force, and Hall force is generated because of the electron’s collision whereas force due to ion slip currents is produced due to the collision of ions. These Ions slip, and Hall forces are in opposite direction to the Lorentz force produced by an applied magnetic field. For the modeling of magnetized partially ionized fluid flow, these forces can be measured by implementing generalized Ohm’s law^28^ with mass, momentum, energy, and Maxwell equations. Qureshi et al*.*^29^ proposed the model of Hall and Ion slip currents-based 3D nano-plasma flow in the presence of thermal radiation. Nazir et al*.*^30^ studied the Cu and Ag nanoparticles based partially ionized Casson nanofluid flow with thermal radiation over a stretching surface. Nawaz et al*.*^31^ investigated the enhancement of thermal properties of partially ionized liquid in the existence of hybrid nanostructures. Nawaz et al*.*^28^ also discussed the comparative computational study of four types of nanoparticles (Fe_3_O_4_, Ag, Al_2_O_3_, TiO_3_) within partially ionized fluid through a stretching sheet.

From the aforesaid literature, it is noted that there is no such study in which Cu/CuO-water and Cu/CuO-kerosene oil partially ionized nanofluids are discussed with CC heat flux model over a linearly stretching surface. A comparison Table 1 is added to divulge the exact novelty of the presented model with the existing available literature. However, due to the high thermal properties of copper and copper-based nanoparticles, our main aim is to analyze the thermal enhancement of two types of nanoparticles Cu and CuO within the two different partially ionized fluids, water and kerosene oil in the existence of CC heat flux model. The numerical methodology is based upon the Finite difference method that is the default in the bvp4c built-in function of the MATLAB scheme. Comparative numerical results are drawn through graphical illustration and tabular form. The whole manuscript is organized into five sections. The modeling and numerical methodology of this study are specified in the second and third sections. Graphical and tabular form results with discussion are represented in section four. Conclusions of the present comparative study are drawn in the last section.

**Table 1:** Literature survey for the uniqueness of the presented mode.

Reference no.	3D model	Cu/CuO-water/kerosene oil	Hall and ion slip
^12^	×	**√**	×
^13^	×	**√**	×
^14^	×	**√**	×
^15^	×	**√**	×
^16^	×	**√**	×
Present	**√**	**√**	**√**

## Mathematical modeling

**Figure 1:**
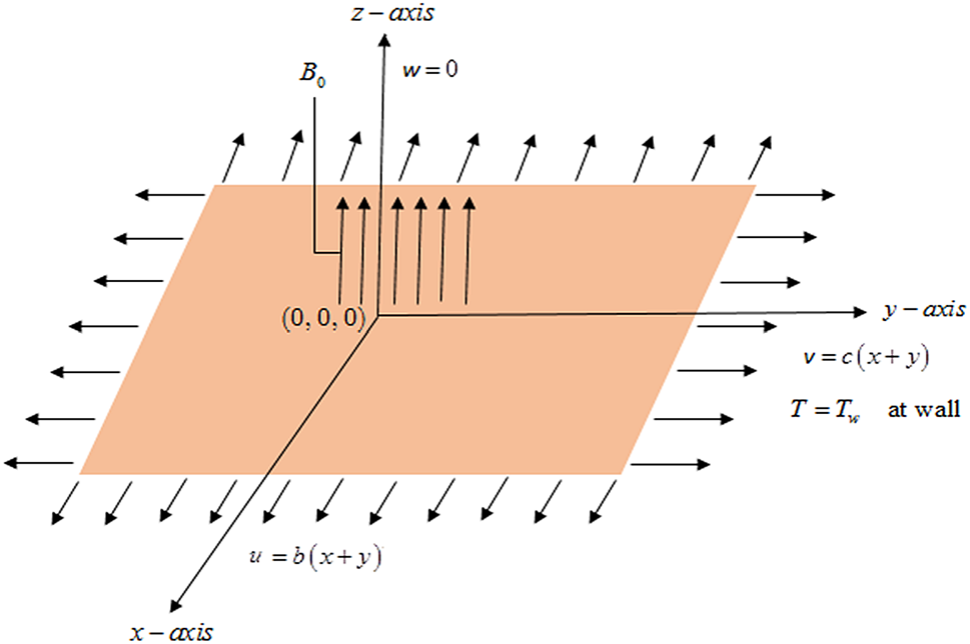
Physical Schematic illustration of the given problem.

We consider 3D Cu/CuO-water and Cu/CuO-kerosene oil partially ionized magnetized nanofluid flow over a horizontal stretching sheet within a constant magnetic field *B*_0_ and CC heat flux. Magnetized partially ionized nanofluid is flowing with the velocity $V_{w} = \left[ {b\left( {x + y} \right),c\left( {x + y} \right),0} \right]$. The geometrical form of our problem is represented in Fig. 1.* T*_*w*_ is the temperature at the surface and *T*_∞_ is the ambient temperature. The effects of viscous and ohmic dissipations are not taken into consideration. As current charges are in motion so there is no applied electric field. The high-velocity flow implies a very small Reynolds number and therefore we have neglected induced magnetic field. MHD (magnetohydrodynamic) equations for incompressible time-independent flow of Newtonian fluid comprising invariable properties are

1 $$\nabla .V = 0,$$

2 $$\rho_{nf} \frac{dV}{{dt}} = - \nabla P + \mu_{nf} \nabla^{2} V + \rho_{nf} \left( {J \times B} \right),$$

$$\nabla .B = 0,\nabla \times E = \frac{\partial B}{{\partial t}},\nabla \times B = \mu_{0} J,$$

3 $$J = \sigma_{nf} \left[ {\frac{{\beta_{H} \beta_{i} }}{{\left| B \right|^{2} }}\left( {J \times B} \right) \times B - \frac{{\beta_{H} }}{\left| B \right|}\left( {J \times B} \right) + \left( {E + V \times B} \right)} \right],$$

with CC heat flux model^14^

4 $$q + \tau_{0} \left\{ {\frac{\partial q}{{\partial t}} + \left( {\nabla .V} \right)q + V.\nabla q - q.\nabla V} \right\} = - \kappa_{nf} \nabla T,$$

5 $$\left( {\rho C_{p} } \right)_{nf} \frac{dT}{{dt}} + \tau_{0} \left\{ {V.\nabla \left( {V.\nabla T} \right)} \right\} = \kappa_{nf} \nabla^{2} T + \frac{1}{{\sigma_{nf} }}J.J.$$

Equations (1) and (2) are the continuity, momentum equations and we get energy Eq. (5) using CC heat flux model Eq. (4). Invoking boundary layer approximations for 3D incompressible steady fluid flow, above Eqs. (1), (2), and (5) reduce to

6 $$\frac{\partial u}{{\partial x}} + \frac{\partial v}{{\partial y}} + \frac{\partial w}{{\partial z}} = 0,$$

7 $$\rho_{nf} \left( {u\frac{\partial u}{{\partial x}} + v\frac{\partial u}{{\partial y}} + w\frac{\partial u}{{\partial z}}} \right) = \mu_{nf} \frac{{\partial^{2} u}}{{\partial z^{2} }} - \frac{{\sigma_{nf} B_{0}^{2} }}{{\left[ {\left( {1 + \beta_{H} \beta_{i} } \right)^{2} + \beta_{H}^{2} } \right]}}\left[ {\left( {1 + \beta_{H} \beta_{i} } \right)u - \beta_{H} v} \right],$$

8 $$\rho_{nf} \left( {u\frac{\partial v}{{\partial x}} + v\frac{\partial v}{{\partial y}} + w\frac{\partial v}{{\partial z}}} \right) = \mu_{nf} \frac{{\partial^{2} v}}{{\partial z^{2} }} - \frac{{\sigma_{nf} B_{0}^{2} }}{{\left[ {\left( {1 + \beta_{H} \beta_{i} } \right)^{2} + \beta_{H}^{2} } \right]}}\left[ {\left( {1 + \beta_{H} \beta_{i} } \right)v + \beta_{H} u} \right],$$

9 $$\begin{gathered} \left( {\rho C_{p} } \right)_{nf} \left( {u\frac{\partial T}{{\partial x}} + v\frac{\partial T}{{\partial y}} + w\frac{\partial T}{{\partial z}}} \right) \hfill \\ + \tau_{0} \left\{ \begin{gathered} u^{2} \frac{{\partial^{2} T}}{{\partial x^{2} }} + v^{2} \frac{{\partial^{2} T}}{{\partial y^{2} }} + w^{2} \frac{{\partial^{2} T}}{{\partial z^{2} }} + 2uv\frac{{\partial^{2} T}}{\partial x\partial y} + 2vw\frac{{\partial^{2} T}}{\partial y\partial z} + 2uw\frac{{\partial^{2} T}}{\partial x\partial z} \hfill \\ +\ \left( {u\frac{\partial u}{{\partial x}} + v\frac{\partial u}{{\partial y}} + w\frac{\partial u}{{\partial z}}} \right)\frac{\partial T}{{\partial x}} + \left( {u\frac{\partial v}{{\partial x}} + v\frac{\partial v}{{\partial y}} + w\frac{\partial v}{{\partial z}}} \right)\frac{\partial T}{{\partial y}} \hfill \\ + \,\left( {u\frac{\partial w}{{\partial x}} + v\frac{\partial w}{{\partial y}} + w\frac{\partial w}{{\partial z}}} \right)\frac{\partial T}{{\partial z}} \hfill \\ \end{gathered} \right\} \hfill \\ = k_{nf} \frac{{\partial^{2} T}}{{\partial z^{2} }}. \hfill \\ \end{gathered}$$

The following BC’s are used to interpret the above problem

10 $$\begin{gathered} u = b\left( {x + y} \right),v = c\left( {x + y} \right),w = 0,T = T_{w} \;{\text{at}}\;z = 0, \hfill \\ u \to 0,v \to 0,w = 0,T \to T_{\infty } \quad {\text{as}}\quad z \to \infty , \hfill \\ \end{gathered}$$

Table 2 represents the mathematical forms of density, heat capacity, dynamic viscosity, kinematic viscosity, thermal conductivity, and electrical thermal conductivity. Table 3 depicts the thermophysical features of the involved base fluids *i.e.,* water, kerosene oil, and the nanoparticles i.e.*,* copper & copper oxide.

**Table 2:** The models for nanofluid thermophysical properties^37,38^.

Properties	Cu-water/CuO-water/Cu-kerosene oil/CuO-kerosene oil
Density	$\rho_{nf} = \phi \rho_{n} + \left( {1 - \phi } \right)\rho_{f}$
Heat capacity	$\left( {\rho C_{p} } \right)_{nf} = \phi \left( {\rho C_{p} } \right)_{n} + \left( {1 - \phi } \right)\left( {\rho C_{p} } \right)_{f}$
Dynamic viscosity	$\mu_{nf} = \frac{{\mu_{f} }}{{\left( {1 - \phi } \right)^{2.5} }}$
Kinematic viscosity	$\nu_{nf} = \frac{{\mu_{nf} }}{{\rho_{nf} }}$
Thermal conductivity	$\alpha_{nf} = \frac{{k_{nf} }}{{\left( {\rho C_{p} } \right)_{nf} }},k_{nf} = \frac{{k_{n} + 2k_{f} - 2\phi \left( {k_{f} - k_{n} } \right)}}{{k_{n} + 2k_{f} + \phi \left( {k_{f} - k_{n} } \right)}}k_{f}$
Electrical thermal conductivity	$\sigma_{nf} = \left\{ {1 + \frac{{3\left( {\frac{{\sigma_{n} }}{{\sigma_{f} }} - 1} \right)\phi }}{{\frac{{\sigma_{n} }}{{\sigma_{f} }} + 2 - \left( {\frac{{\sigma_{n} }}{{\sigma_{f} }} - 1} \right)\phi }}} \right\}$

**Table 3:** Mathematical values of thermal properties of Cu, CuO, water, and kerosene oil^32–35^.

Thermophysical properties	$\rho \left( {{\text{kg}}\;{\text{m}}^{{3}} } \right)$	$C_{p} \left( {{\text{J}}\;{\text{kg}}^{ - 1} {\text{K}}^{ - 1} } \right)$	$k\left( {{\text{W}}\;{\text{m}}^{ - 1} {\text{K}}^{ - 1} } \right)$	$\sigma \left( {{\text{s}}\;{\text{m}}^{ - 1} } \right)$
Cu	8933	385	401	5.96 × 10^7^
CuO	6500	540	18	2.7 × 10^–8^
Water	997.1	4179	0.13	5.5 × 10^–5^
Kerosene oil	783	2090	0.15	21 × 10^–6^

## Similarity transformations

To convert a highly nonlinear dimensional system of equations into an ordinary linear non-dimensional system of equations, the change of variables is given as

11 $$\begin{gathered} u = b\left( {x + y} \right)f^{\prime}\left( \eta \right),v = b\left( {x + y} \right)g^{\prime}\left( \eta \right),\eta = \left( {\frac{b}{{\nu_{f} }}} \right)^{\frac{1}{2}} z, \hfill \\ w = - \left( {b\nu_{f} } \right)^{\frac{1}{2}} \left[ {f\left( \eta \right) + g\left( \eta \right)} \right],T = \left( {T_{w} - T_{\infty } } \right)\theta \left( \eta \right) + T_{\infty } . \hfill \\ \end{gathered}$$

Using the above change of variables, the continuity equation is trivially satisfied, and Eqs. (7)– (10) take the form

12 $$\begin{gathered} \left( {\frac{1}{{\phi_{1} }}} \right)f^{\prime\prime\prime}\left( \eta \right) + f^{\prime\prime}\left( \eta \right)\left[ {f\left( \eta \right) + g\left( \eta \right)} \right] = f^{\prime}\left( \eta \right)\left[ {f^{\prime}\left( \eta \right) + g^{\prime}\left( \eta \right)} \right] \hfill \\ +\, \left( {Ha} \right)^{2} \left( {\frac{{\phi_{2} }}{{\phi_{1} }}} \right)\left\{ {\frac{{\left( {1 + \beta_{H} \beta_{i} } \right)f^{\prime}\left( \eta \right) - \beta_{H} g^{\prime}\left( \eta \right)}}{{\left( {1 + \beta_{H} \beta_{i} } \right)^{2} + \beta_{H}^{2} }}} \right\}, \hfill \\ \end{gathered}$$

13 $$\begin{gathered} \left( {\frac{1}{{\phi_{1} }}} \right)g^{\prime\prime\prime}\left( \eta \right) + g^{\prime\prime}\left( \eta \right)\left[ {f\left( \eta \right) + g\left( \eta \right)} \right] = g^{\prime}\left( \eta \right)\left[ {f^{\prime}\left( \eta \right) + g^{\prime}\left( \eta \right)} \right] \hfill \\ +\, \left( {Ha} \right)^{2} \left( {\frac{{\phi_{2} }}{{\phi_{1} }}} \right)\left[ {\frac{{\left( {1 + \beta_{H} \beta_{i} } \right)g^{\prime}\left( \eta \right) + \beta_{H} f^{\prime}\left( \eta \right)}}{{\left( {1 + \beta_{H} \beta_{i} } \right)^{2} + \beta_{H}^{2} }}} \right], \hfill \\ \end{gathered}$$

14 $$\left( {\frac{1}{{\phi_{3} }}\frac{{k_{nf} }}{{k_{f} }}} \right)\theta^{\prime\prime}\left( \eta \right) = \Pr \left[ \begin{gathered} \gamma \left\{ \begin{gathered} \left( {f\left( \eta \right) + g\left( \eta \right)} \right)^{2} \theta^{\prime\prime}\left( \eta \right) \hfill \\ + \left( {f\left( \eta \right) + g\left( \eta \right)} \right)\left( {f^{\prime}\left( \eta \right) + g^{\prime}\left( \eta \right)} \right)\theta^{\prime}\left( \eta \right) \hfill \\ \end{gathered} \right\} \hfill \\ - \left( {f\left( \eta \right) + g\left( \eta \right)} \right)\theta^{\prime}\left( \eta \right) \hfill \\ \end{gathered} \right],$$

with transformed BC’s

15 $$\begin{gathered} f^{\prime}\left( 0 \right) = 1,f\left( 0 \right) = 0,g^{\prime}\left( 0 \right) = a,g\left( 0 \right) = 0,\theta \left( 0 \right) = 1\quad {\text{at}}\quad \eta = 0, \hfill \\ f^{\prime}\left( \infty \right) \to 0,g^{\prime}\left( \infty \right) \to 0,\theta \left( \infty \right) \to 0\quad {\text{as}}\quad \eta \to \infty \hfill \\ \end{gathered}$$

where

16 $$\begin{gathered} \phi_{1} = \left( {1 - \phi } \right)^{2.5} \left( {1 + \phi - \phi \frac{{\rho_{n} }}{{\rho_{f} }}} \right), \hfill \\ \phi_{2} = \left( {1 - \phi } \right)^{2.5} \left\{ {1 + \frac{{3\left( {\frac{{\sigma_{n} }}{{\sigma_{f} }} - 1} \right)\phi }}{{\frac{{\sigma_{n} }}{{\sigma_{f} }} + 2 - \left( {\frac{{\sigma_{n} }}{{\sigma_{f} }} - 1} \right)\phi }}} \right\}, \hfill \\ \phi_{3} = \left( {1 + \phi - \phi \frac{{\left( {\rho C_{p} } \right)_{n} }}{{\left( {\rho C_{p} } \right)_{f} }}} \right), \hfill \\ \end{gathered}$$

Here, prime denotes the partial derivative with respect to $\eta$. The expressions for the non-dimensional parameters (given in Eqs. (12)–(15)) are:

17 $$a = \frac{c}{b},Ha = \sqrt {\frac{{\sigma_{nf} B_{0}^{2} }}{{b\rho_{f} }}} ,\Pr = \frac{{\mu_{f} \left( {C_{p} } \right)_{{_{f} }} }}{{k_{f} }},\gamma = \tau_{0} b.$$

Mathematically, dimensional forms of Skin drag coefficient and Nusselt number are:

18 $$\begin{gathered} C_{x} = \frac{{\tau_{zx} }}{{\rho_{f} u^{2} }},C_{y} = \frac{{\tau_{zy} }}{{\rho_{f} v^{2} }},Nu_{x} = \frac{{\left( {x + y} \right)q_{w} }}{{k_{f} \left( {T_{w} - T_{\infty } } \right)}}, \hfill \\ \tau_{zx} = \mu_{nf} \left( {u_{z} + w_{x} } \right)_{z = 0} ,\tau_{zy} = \mu_{nf} \left( {v_{z} + w_{y} } \right)_{z = 0} . \hfill \\ \end{gathered}$$

Implementing Eq. (11), we get a non-dimensional form of Nusselt and Skin drag coefficient given in Eq. (18) as:

19 $$\begin{gathered} \left( {{\text{Re}}_{{x_{L} }} } \right)^{0.5} C_{x} = \frac{1}{{\phi_{1} }}f^{^{\prime\prime}} \left( 0 \right), \hfill \\ \left( {{\text{Re}}_{{y_{L} }} } \right)^{0.5} C_{y} = \frac{1}{{\phi_{1} }}g^{^{\prime\prime}} \left( 0 \right), \hfill \\ \left( {{\text{Re}}_{{x_{L} }} } \right)^{0.5} Nu_{x} = - \frac{{k_{nf} }}{{k_{f} }}\theta^{^{\prime}} \left( 0 \right), \hfill \\ \end{gathered}$$

where ${\text{Re}}_{{x_{L} }}$ and ${\text{Re}}_{{y_{L} }}$ is the local Reynolds number given as

20 $${\text{Re}}_{{x_{L} }} = \frac{{c\left( {x + y} \right)}}{{\nu_{f} }},{\text{Re}}_{{y_{L} }} = \frac{{c\left( {x + y} \right)}}{{\nu_{f} }}.$$

## Solution methodology

For non-linear systems of ODEs (Eqs. 12–14) with boundary conditions (Eq. 16), the Finite-difference default method of bvp4c built-in function of MATLAB scheme is implemented which is fourth-order accurate and 0.01 grid size is taken with the tolerance 10^–6^. Using the following numerical code, we get ODEs with order one.

21 $$\begin{gathered} y\left( 1 \right) = f\left( \eta \right), \hfill \\ y\left( 2 \right) = f^{\prime}\left( \eta \right), \hfill \\ y\left( 3 \right) = f^{\prime\prime}\left( \eta \right), \hfill \\ f^{\prime\prime\prime}\left( \eta \right) = y^{\prime}\left( 3 \right) = yy\left( 1 \right) = \phi_{1} \left[ \begin{gathered} \left\{ {y\left( 2 \right) + y\left( 5 \right)} \right\}y\left( 2 \right) - \left\{ {y\left( 1 \right) + y\left( 4 \right)} \right\}y\left( 3 \right) \hfill \\ + \left( {\frac{{\phi_{2} }}{{\phi_{1} }}} \right)\left( {Ha} \right)^{2} \left\{ {\frac{{\left( {1 + \beta {}_{H}\beta_{i} } \right)y\left( 2 \right) - \beta {}_{H}y\left( 5 \right)}}{{\left( {1 + \beta {}_{H}\beta_{i} } \right)^{2} + \beta_{H}^{2} }}} \right\} \hfill \\ \end{gathered} \right], \hfill \\ \end{gathered}$$

22 $$\begin{gathered} y\left( 4 \right) = g\left( \eta \right), \hfill \\ y\left( 5 \right) = g^{\prime}\left( \eta \right), \hfill \\ y\left( 6 \right) = g^{\prime\prime}\left( \eta \right), \hfill \\ g^{\prime\prime\prime}\left( \eta \right) = y^{\prime}\left( 6 \right) = yy\left( 2 \right) = \phi_{1} \left[ \begin{gathered} \left\{ {y\left( 2 \right) + y\left( 5 \right)} \right\}y\left( 5 \right) - \left\{ {y\left( 1 \right) + y\left( 4 \right)} \right\}y\left( 6 \right) \hfill \\ + \left( {\frac{{\phi_{2} }}{{\phi_{1} }}} \right)\left( {Ha} \right)^{2} \left\{ {\frac{{\left( {1 + \beta {}_{H}\beta_{i} } \right)y\left( 5 \right) + \beta {}_{H}y\left( 2 \right)}}{{\left( {1 + \beta {}_{H}\beta_{i} } \right)^{2} + \beta_{H}^{2} }}} \right\} \hfill \\ \end{gathered} \right], \hfill \\ \end{gathered}$$

23 $$\begin{gathered} y\left( 7 \right) = \theta \left( \eta \right), \hfill \\ y\left( 8 \right) = \theta^{^{\prime}} \left( \eta \right), \hfill \\ g^{\prime\prime}\left( \eta \right) = y^{\prime}\left( 6 \right) = yy\left( 3 \right) = \frac{{\Pr \left[ \gamma \left\{ {y\left( 1 \right) + y\left( 4 \right)} \right\}\left\{ {y\left( 2 \right) + y\left( 5 \right)} \right\}y\left( 8 \right) \right]}}{{\frac{1}{{\phi_{3} }}\frac{{k_{nf} }}{{k_{f} }} - \Pr \gamma \left\{ {y\left( 1 \right) + y\left( 4 \right)} \right\}^{2} }}, \hfill \\ \end{gathered}$$

with the boundary conditions

24 $$\begin{gathered} y0\left( 2 \right) = 1,y0\left( 5 \right) = a,y0\left( 1 \right) = 0,y0\left( 4 \right) = 0,y0\left( 7 \right) = 1, \hfill \\ y\inf \left( 2 \right) = 0,y\inf \left( 5 \right) = 0,y\inf \left( 7 \right) = 0. \hfill \\ \end{gathered}$$

## Results and discussion

The heat transfer effects of Cu and CuO nanoparticles in water and kerosene oil based partially ionized magnetized nanofluids in the presence of CC heat flux model are analyzed theoretically. The mathematical system of equations is solved via numerical default method of bvp4c built-in function of the MATLAB scheme which is a fourth-order accurate method. The different effects are simulated numerically and presented in both graphical and tabular forms. Fluid flows in both *x*- and *y-*directions, shear stresses, and rate of heat transfer at the wall are investigated under some prominent physical parameters. The parameters used in this analysis are $a = 0.5,\Pr = 7,\phi = 0.01,\gamma = 0.5,Ha = 0.8,\beta_{i} = 0.5,$ $\beta_{H} = 0.3.$

### Flow behavior of copper and copper-based partially ionized water and kerosene oil nanofluids

**Figure 2:**
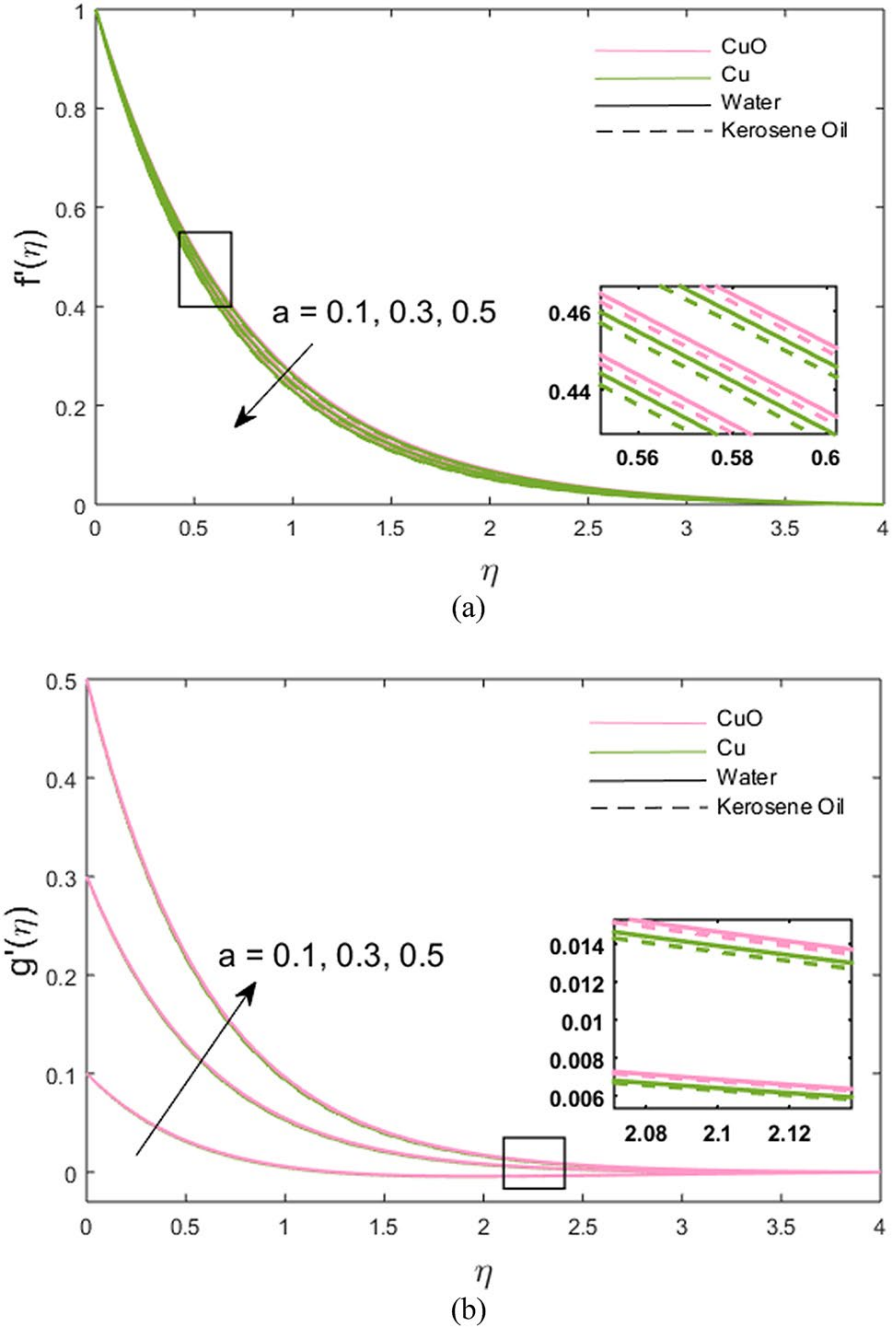
**(a,b)** Variations of stretching rate *a* on velocity in *x*- and *y*-direction.

**Figure 3:**
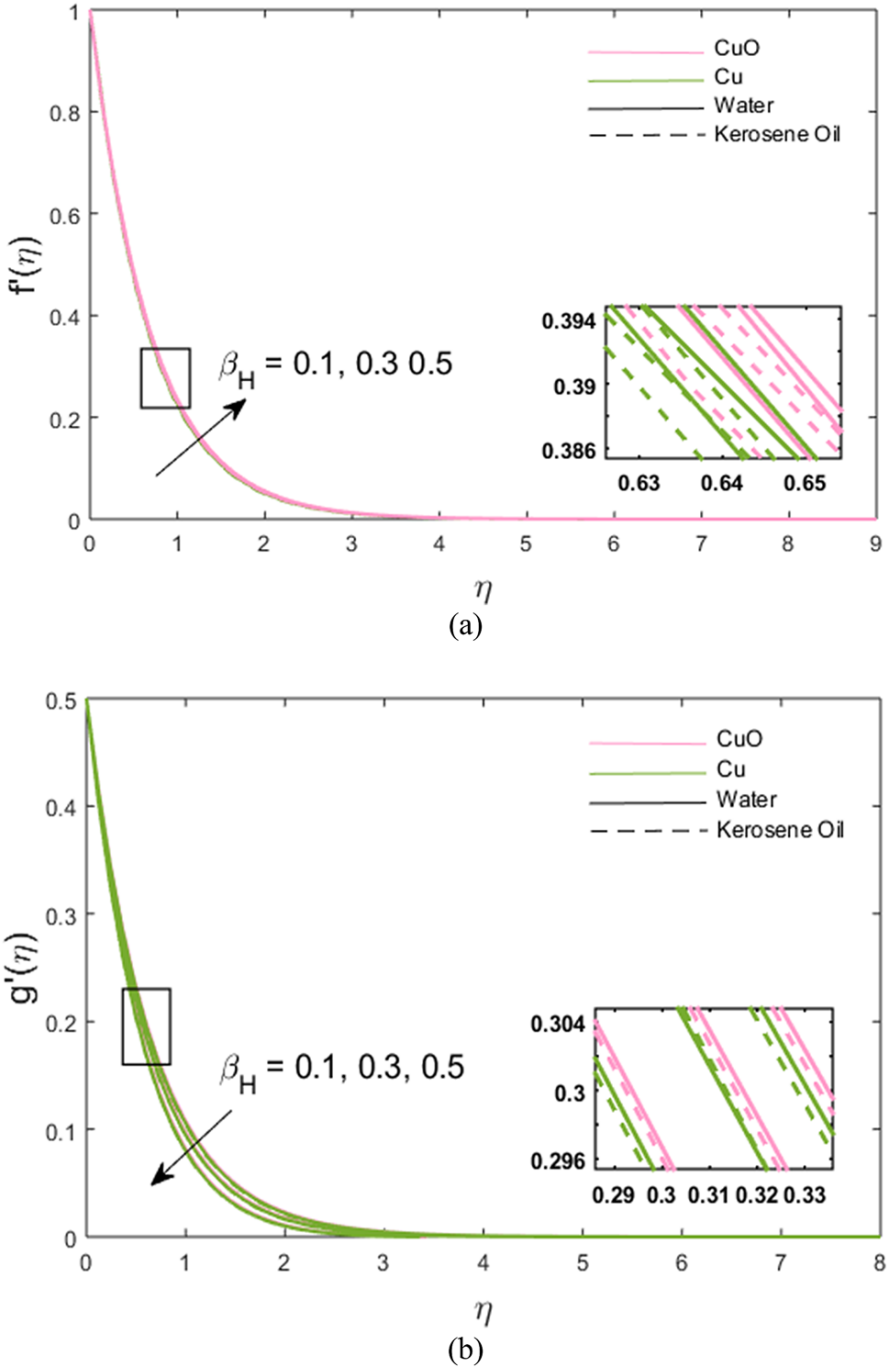
**(a,b)** Variations of Hall parameter $\beta_{H}$ on velocity in *x*- and *y*-direction.

**Figure 4:**
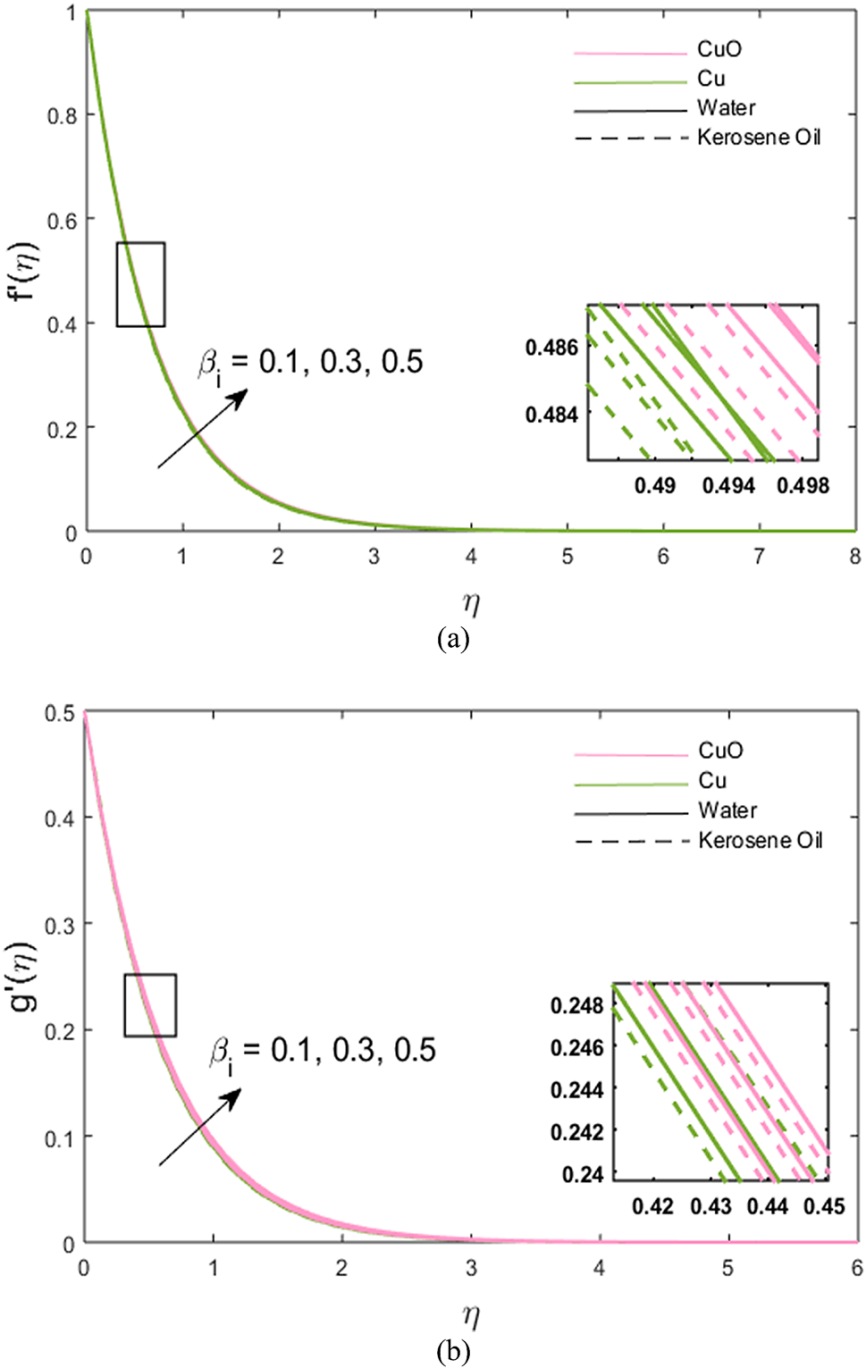
**(a,b)** Variations of ion slip parameter $\beta_{i}$ on velocity in *x*- and *y*-direction.

**Figure 5:**
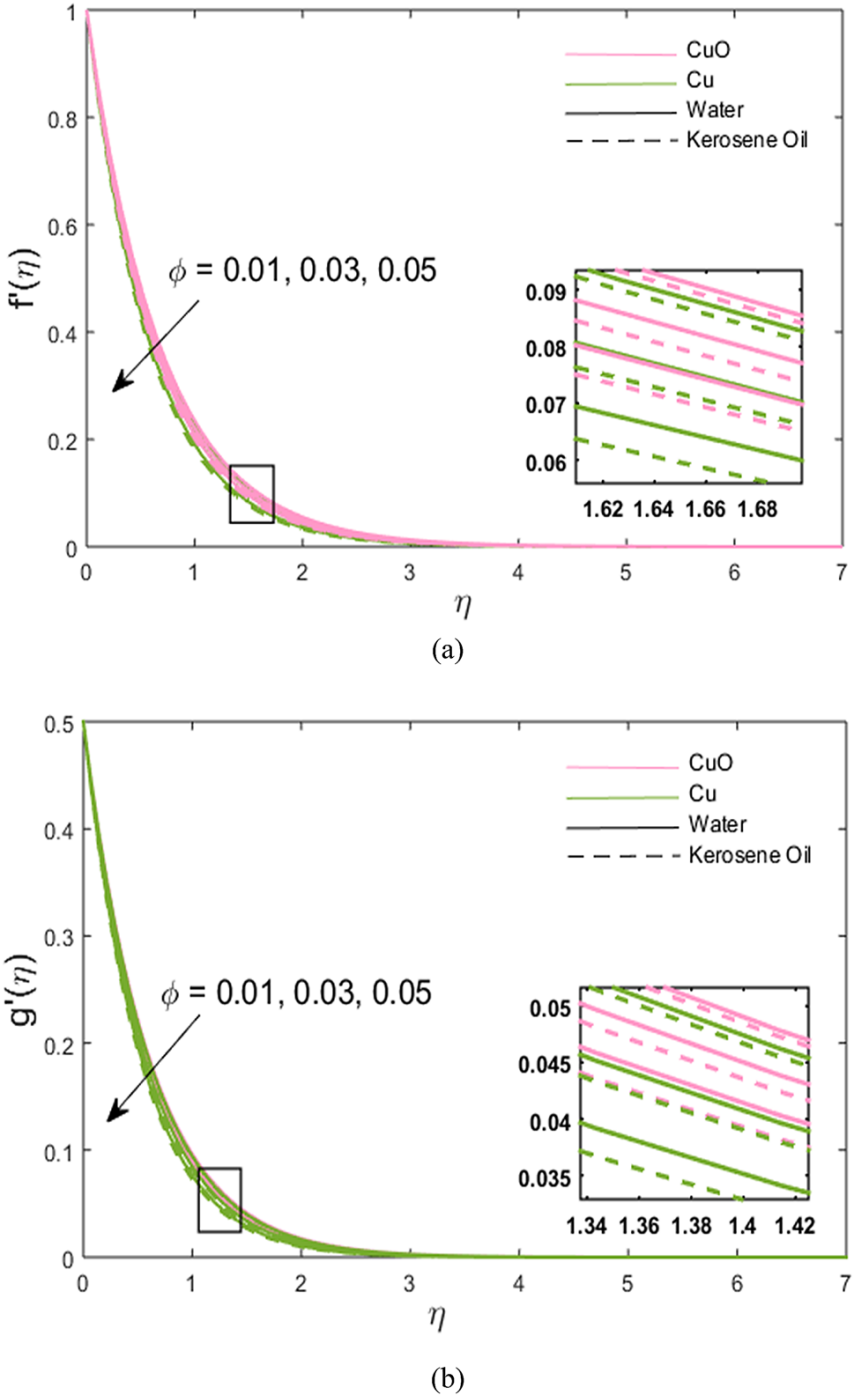
**(a,b)** Variations of nanoparticle volume fraction $\phi$ on velocity in *x*- and *y*-direction.

The impacts of stretching ratio parameter $\left( a \right)$, Hall parameter $\left( {\beta_{H} = \omega_{H} \tau_{H} } \right)$, ion slip parameter $\left( {\beta_{i} = \omega_{i} \tau_{i} } \right)$, and nanoparticles volume fraction $\left( \phi \right)$ on fluid velocity in *x-* and *y*-directions are presented graphically in Figs. 2, 3, 4, 5. Figure 2a,b show the variations of $\left( {a = {c \mathord{\left/ {\vphantom {c b}} \right. \kern-\nulldelimiterspace} b}} \right)$ on *x*- and *y*-velocity components which are the ratio between the rate of stretching surface *c* in the *y*-direction and the rate of stretching *d* in the *x*-direction. Hence, it is concluded that velocity increases towards the *y*-direction and decreases towards the *x*-direction. In *y*-direction, the momentum diffusion is faster than the momentum diffusion in the *x-*direction. It is noted that the velocity of partially ionized nanofluid flow is higher in the case of CuO-water nanofluid such that the velocity of CuO-water nanofluid > CuO-kerosene oil nanofluid > Cu-water nanofluid > Cu-kerosene oil nanofluid. In Figs. 3a,b, the *x*-component of velocity enhances due to increment in $\left( {\beta_{H} } \right)$ and *y*-component of velocity falls. The behavior of $\beta_{i} \left( { = \tau_{i} \omega_{i} } \right)$ on *x-* and *y-*velocity components are drawn in Fig. 4a,b. The fluid velocity in both *x* and *y* directions is noted to increase with the augmentation in the Ion slip parameter. It is also observed that the effect of $\left( {\beta_{i} } \right)$ on *x-*velocity component is analogous to the $\left( {\beta_{H} } \right)$ on the *x-*velocity component of the partially ionized fluid. Since an enhancement in the fluid motion is noted due to time collision of ions $\left( {\tau_{i} } \right)$ or ions frequency $\left( {\omega_{i} } \right)$ caused by an increment in ion slip parameter, hence; velocity increases. Figure 5a,b depict the decreasing behavior of momentum transport in both directions for the high volume of Cu and CuO nanoparticles. As high nanoparticle concentration causes a gradual decrease in a fluid motion. Thus, the velocity decreases. It is again noted that whenever velocity increases, the partially ionized fluid velocity of CuO-water nanofluid is higher than the other nanofluids, the same as discussed for Fig. 2. And, whenever velocity falls, a large decay in fluid velocity is observed in the case of Cu-kerosene oil nanofluid such that the velocity of Cu-kerosene oil nanofluid < Cu-water nanofluid < CuO-kerosene oil nanofluid < CuO-water nanofluid (Figs. 2a, 3b, 5a,b).

### Dynamics of heat transfer

**Figure 6:**
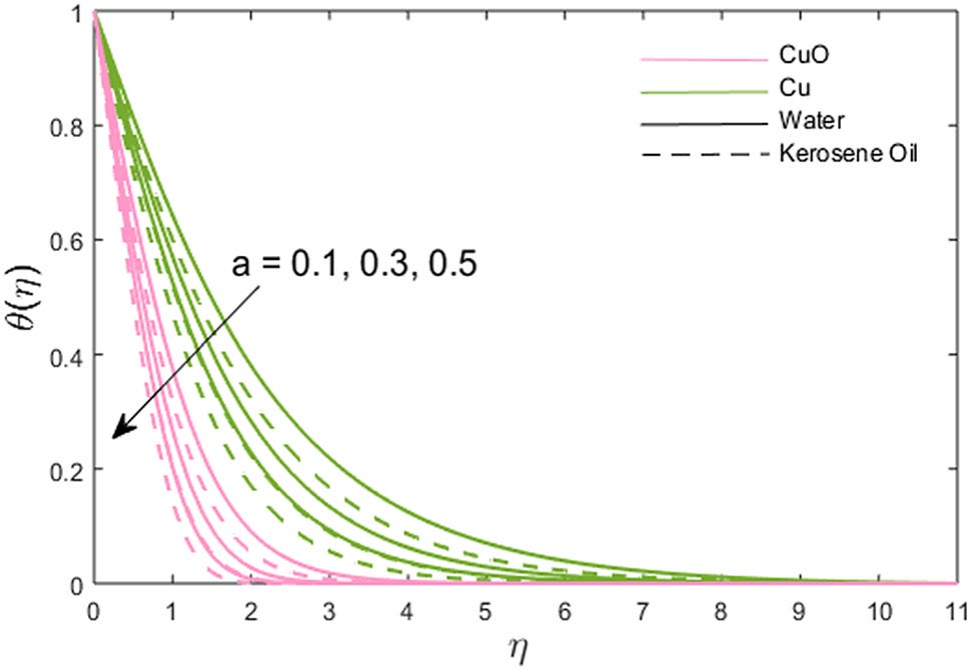
Variations of stretching rate *a* on fluid temperature.

**Figure 7:**
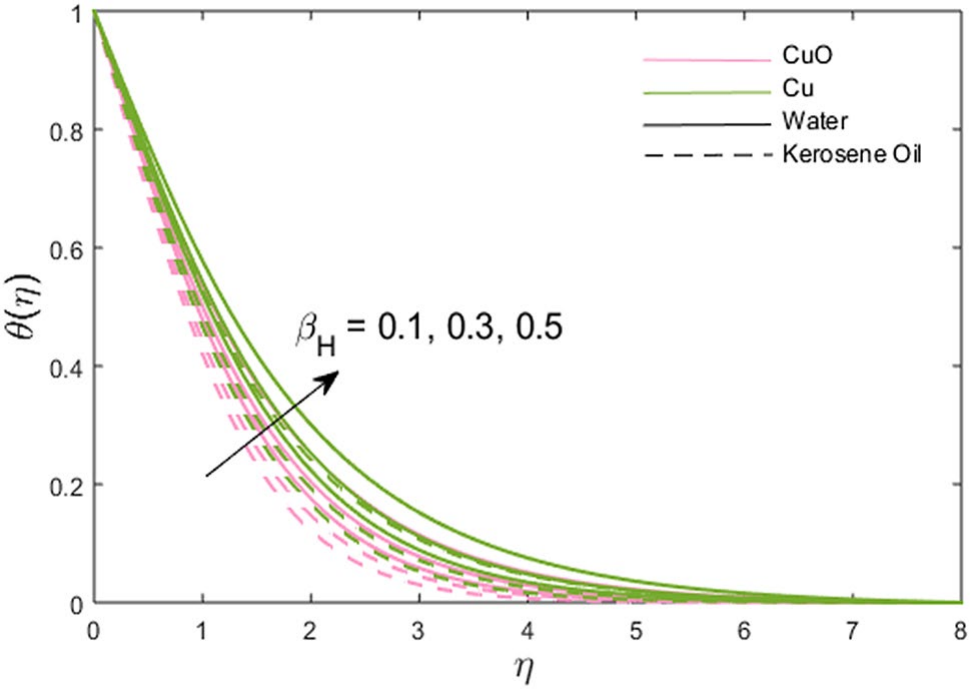
Variations of Hall parameter $\beta_{H}$ on fluid temperature.

**Figure 8:**
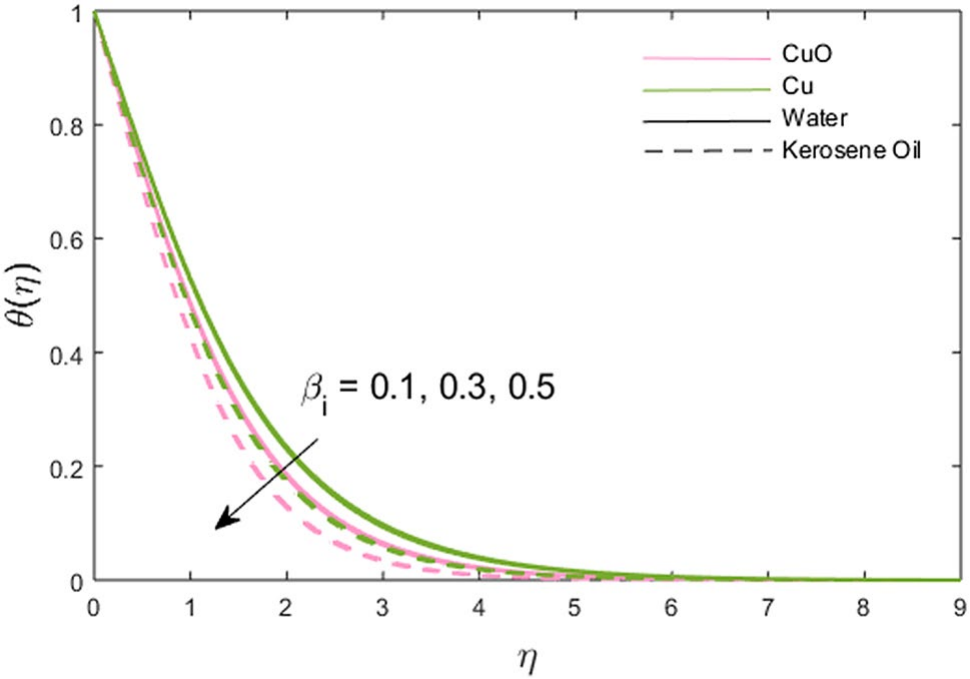
Variations of ion slip parameter $\beta_{i}$ on fluid temperature.

**Figure 9:**
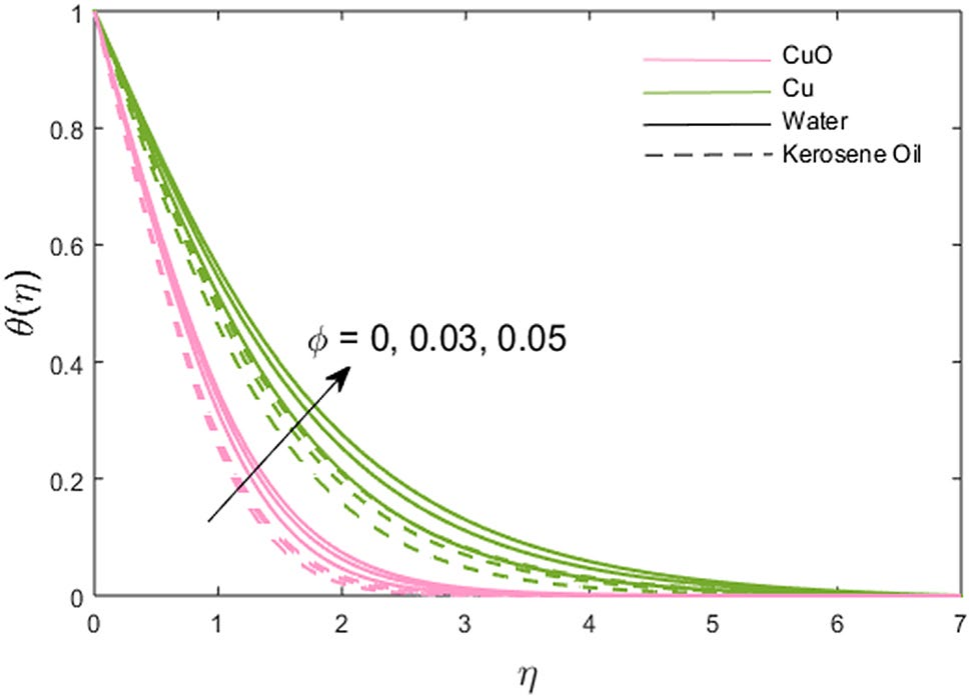
Variations of nanoparticle volume fraction $\phi$ on fluid temperature.

**Figure 10:**
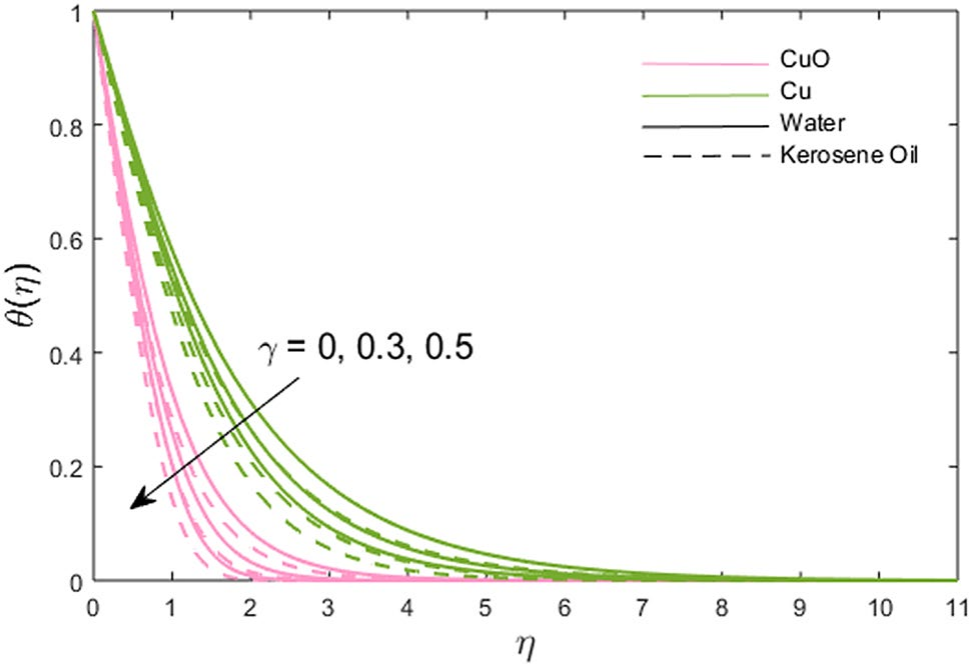
Variations of thermal time relaxation parameter $\gamma$ on fluid temperature.

The impacts of the stretching ratio parameter $\left( a \right)$, Hall parameter $\left( {\beta_{H} = \omega_{H} \tau_{H} } \right)$, Ion slip parameter $\left( {\beta_{i} } \right)$, nanoparticles volume fraction $\left( \phi \right)$, and thermal time relaxation parameter $\left( \gamma \right)$ on partially the ionized fluid temperature in *x-* and *y*-direction for linear stretching surface are presented graphically in Figs. 6, 7, 8, 9, 10. Figure 6 shows the decreasing trend of stretching ratio parameter on fluid temperature. It is noted that temperature variations are higher for Cu-water and Cu-kerosene oil nanofluids than the CuO-water and CuO-kerosene oil nanofluids. In Figs. 7 and 8, the influence of the Hall and Ion slip parameter is simulated on partially ionized fluid temperature. An increment in these parameters has a decreasing trend on the partially ionized fluid temperature in case of $\beta_{i}$ whereas the increasing trend in case of $\beta_{H}$. The dynamics of fluid temperature for nanoparticles volume fraction $\phi$ are illustrated in Fig. 9. Two different types of nanoparticles are dispersed in the two different types of partially ionized liquids. The thermal conductivity effectiveness is increased due to nanoparticle dispersion in the mixture. The greater effective thermal conductivity is noted for the mixture of Cu-nanoparticles and base fluid water as compared to other partially ionized nanofluids (Cu-kerosene oil, CuO-water/kerosene oil partially ionized nanofluids). Thus, it is concluded that the dispersion of CuO-nanoparticles in fluid other than in partially ionized fluid is recommended for greater thermal conductance. The observations for partially ionized fluid temperature under the variations of thermal time relaxation parameter $\gamma$ are sketched in Fig. 10. Partially ionized nanofluid temperature is reduced under the higher values of $\gamma$. Besides, the zero thermal relaxation time narrates to traditional Fourier's law, so this can be deduced that the temperature is smaller than the classical Fourier's model. It is observed under the effects of prominent parameters that the temperature is highest when Cu-nanoparticles are dispersed in the partially ionized water-base fluid than the other three given partially ionized nanofluids.

### Normalized shear stresses and heat flux at the wall

In this part, the effects of different parameters on the rate of heat transfer and skin drag are presented in a tabular form. Numerical values for heat flux and skin drag are tabulated in Table 4 for two types of partially ionized fluids (water and kerosene oil) verses two types of nanoparticles volume fraction $\phi$, Hartmann number $Ha$, thermal time relaxation parameter $\gamma$, Hall and ion slip parameters $\beta_{H}$ and $\beta_{i}$, and stretching ratio parameter $a$. It is observed that shear stress at the wall in *x*-direction decreases with the increment in Hall parameter while shear stress at the wall is increased by augmentation in the ion slip parameter. However, shear stress at the wall in *y*-direction decreases with augmentation in the Hall parameter, whereas shear stress at the wall is increased with the increment in the ion slip parameter. The heat flux at the wall is increased when $\beta_{i}$ is increased. A slight increase in wall heat flux can be seen from numerical results by increasing the thermal time relaxation parameter $\gamma$. Skin friction (both in *x-* and *y-*direction) and heat flux at the wall have an increasing trend when the dispersion of nanoparticles is increased.

**Table 4:** Numerical analysis of Surface drag and Nusselt number for a linearly stretching sheet.

$\phi$	$Ha$	$\gamma$	$\beta_{H}$	$\beta_{i}$	$a$	Water		Kerosene oil	
						Cu	CuO	Cu	CuO
**Skin/surface drag analysis in the** *x-***direction**									
0.01						− 1.3478952	− 1.3612856	− 1.3290680	− 1.3468623
0.02						− 1.2809354	− 1.3045165	− 1.2483745	− 1.2790191
0.03						− 1.2209122	− 1.2522615	− 1.1782113	− 1.2180605
0.01	0.1					− 1.1690065	− 1.1815156	− 1.1558839	− 1.1716080
	0.3					− 1.1931705	− 1.2065709	− 1.1792828	− 1.1960799
	0.5					− 1.2399456	− 1.2528109	− 1.2240452	− 1.2411975
	0.8	0.1				− 1.3478952	− 1.3612856	− 1.3290680	− 1.3471201
		0.3				− 1.3478952	− 1.3612856	− 1.3290680	− 1.3471201
		0.5				− 1.3478952	− 1.3612856	− 1.3290680	− 1.3471201
		0.5	0.1			− 1.3652991	− 1.3787380	− 1.3460022	− 1.3639729
			0.3			− 1.3659952	− 1.3612856	− 1.3290680	− 1.3468623
			0.5			− 1.3670789	− 1.3609839	− 1.3264790	− 1.3443249
			0.3	0.1		− 1.3602233	− 1.3736169	− 1.3410002	− 1.3589029
				0.3		− 1.3537355	− 1.3671276	− 1.3347209	− 1.3525666
				0.5		− 1.3478952	− 1.3612856	− 1.3290680	− 1.3468623
				0.5	0.1	− 1.2289698	− 1.2405280	− 1.2105855	− 1.2267139
					0.3	− 1.2899924	− 1.3024977	− 1.2713945	− 1.2885132
					0.5	− 1.3478952	− 1.3612856	− 1.3290680	− 1.3468623
**Skin/surface drag analysis in the** *y-***direction**									
0.01						**− **0.7481555	**− **0.7549908	− 0.7366010	− 0.7462150
0.02						**− **0.7090244	− 0.7208294	− 0.6891233	− 0.7053459
0.03						− 0.6741528	− 0.6895493	− 0.6481502	− 0.6688613
0.01	0.1					**− **0.5861337	**− **0.5923779	− 0.5795261	− 0.5873872
	0.3					**− **0.6094333	**− **0.6161330	− 0.6020764	− 0.6105802
	0.5					**− **0.6529924	**− **0.6596934	− 0.6442662	− 0.6531216
	0.8	0.1				**− **0.7481555	**− **0.7549908	− 0.7366010	− 0.7463125
		0.3				**− **0.7481555	**− **0.7549908	− 0.7366010	− 0.7463125
		0.5				**− **0.7481555	**− **0.7549908	− 0.7366010	− 0.7463125
		0.5	0.1			− 0.7094796	− 0.7162494	− 0.6990055	− 0.7083001
			0.3			**− **0.7481555	**− **0.7549908	− 0.7366010	− 0.7462150
			0.5			− 0.8043621	− 0.8113016	− 0.7911882	− 0.8013409
			0.3	0.1		**− **0.7690649	**− **0.7759392	− 0.7569085	− 0.7667223
				0.3		**− **0.7578884	**− **0.7647415	− 0.7460530	− 0.7557598
				0.5		− 0.7481555	**− **0.7549908	− 0.7366010	− 0.7462150
				0.5	0.1	**− **0.1887964	**− **0.1900994	− 0.1851113	− 0.1873405
					0.3	**− **0.4546673	**− **0.4585576	− 0.4471607	− 0.4529255
					0.5	**− **0.7481554	**− **0.7549908	− 0.7366010	− 0.7462150
**Rate of heat transfer analysis**									
0.01						7.62570480	6.74610810	7.39028910	6.77532020
0.02						7.40457490	6.56191800	7.19080400	6.64042350
0.03						7.20182010	6.38516550	7.01120300	6.51230990
0.01	0.1					8.84073250	8.09590100	8.64775540	8.09759640
	0.3					8.56542160	7.80225640	8.35315670	7.81075800
	0.5					8.24273840	7.44828030	8.02400550	7.46420840
	0.8	0.1				7.62423740	6.68397280	7.38823010	6.70226120
		0.3				7.62497060	6.71482800	7.38925900	6.73769510
		0.5				7.62570480	6.74610810	7.39028910	6.77532020
		0.5	0.1			7.69683580	6.82813970	7.46333790	6.85591820
			0.3			7.62570480	6.74610810	7.39028910	6.77532020
			0.5			7.43556690	6.52417060	7.19450440	6.55655470
			0.3	0.1		7.53355578	6.63861360	7.29534810	6.66929950
				0.3		7.58225670	6.69550890	7.34553480	6.72542900
				0.5		7.62570480	6.74610810	7.39028910	6.77532020
				0.5	0.1	6.51155540	5.34888110	6.22776550	5.37534120
					0.3	7.08742720	6.08678460	6.82890480	6.10761100
					0.5	7.62570480	6.74610810	7.39028910	6.77532020

## Conclusion

Three-dimensional comparative heat transfer analysis of two different partially ionized fluids (water and kerosene oil) using two different nanoparticles (Cu and CuO) over a three-dimensional stretching sheet is studied theoretically. The present theoretical study has depicted that the effects of Cu-nanoparticles are more significant than the CuO-nanoparticles on temperature of partially ionized nanofluid. It is important to mention that the temperature is highest under the effects of prominent parameters when Cu-nanoparticles are dispersed in the partially ionized water base fluid than the other three given partially ionized nanofluids. It is again noted that whenever velocity increases, the partially ionized fluid velocity of CuO-water nanofluid is higher than the other nanofluids and whenever velocity falls, a large decay in fluid velocity is observed in the case of Cu-kerosene oil nanofluid. The impact of Hall parameter $\beta_{H}$ on velocity field in the *y*-direction is more significant as compared to the velocity field in *x-* direction. The greater effective thermal conductivity is noted for Cu-water partially ionized nanofluid as compared to other given partially ionized nanofluids (Cu-kerosene oil, CuO-water/kerosene oil partially ionized nanofluids). Thus, it is concluded that the dispersion of CuO-nanoparticles in base fluid kerosene oil other than in partially ionized water fluid is recommended for greater thermal conductance.

## Abbreviations

CC: Cattaneo–Christov
Nu_x_: Local Nusselt number

## Symbols

$b,c$: Positive dimensional constants
$C_{x}$: Skin friction coefficient in the *x-*direction (dimensionless)
$C_{y}$: Skin friction coefficient in the *y-*direction (dimensionless)
$C_{p}$: Specific heat capacity $\left( {{\text{J kg}}^{{ - 1}} {\text{ K}}^{{ - 1}} } \right)$
$\left( {C_{p} } \right)_{f}$: Specific heat capacity of fluid (–)
$\left( {C_{p} } \right)_{nf}$: Specific heat capacity of nanofluid (–)
$\left( {C_{p} } \right)_{n}$: Specific heat capacity of nanoparticles (–)
$f^{\prime}\left( \eta \right)$: Dimensionless velocity in *the x-*direction
$g^{\prime}\left( \eta \right)$: Dimensionless velocity in the *y-*direction
$Ha$: Hartmann number (Dimensionless)
$$k$$: Thermal conductivity $$\left( {{\text{W K}}^{ - 1} {\text{m}}^{ - 1} } \right)$$
$k_{n}$: Thermal conductivity of nanoparticles (–)
$k_{f}$: Thermal conductivity of the fluid (–)
$k_{nf}$: Thermal conductivity of nanofluid (–)
$\Pr$: Prandtl number (dimensionless)
${\text{Re}}_{{x_{L} }} ,{\text{Re}}_{{y_{L} }}$: Local Reynolds number in *x* and *y*-direction (dimensionless)
$$T$$: Temperature $$\left( {\text{K}} \right)$$
$T_{w}$: Temperature of surface (–)
$$T_{\infty }$$: Ambient temperature (–)
$u,v,w$: Components of Velocity in *x-,y-,z-*directions $\left( {{\text{m s}}^{ - 1} } \right)$
$$x,y,z$$: Axis coordinates $$\left( {\text{m}} \right)$$
$q_{w}$: Heat flux near the wall
$q$: Heat flux vector
$J$: Current density vector
$B$: Magnetic induction vector
$E$: Electric induction vector

## Greek symbols

$\eta$: Similarity transformation variable (dimensionless)
$$\theta \left( \eta \right)$$: Dimensionless temperature
$$\rho$$: Density $$\left( {{\text{kg m}}^{ - 3} } \right)$$
$\rho_{n}$: Density of nanoparticles (–)
$\rho_{f}$: Density of fluid (–)
$\rho_{nf}$: Density of nanofluid (–)
$$\mu$$: Dynamic viscosity $$\left( {{\text{kg m}}^{ - 1} {\text{s}}^{ - 1} } \right)$$
$\mu_{f}$: Fluid dynamic viscosity (–)
$\mu_{nf}$: Nanofluid dynamic viscosity (–)
$\phi$: Volume fraction of nanoparticles $\left( {{\text{mol L}}^{ - 1} } \right)$
$\sigma_{f}$: Electrical conductivity of fluid $\left( {{\text{kg m}}^{ - 3} {\text{s}}^{3} {\text{A}}^{2} } \right)$
$\sigma_{nf}$: Nanofluid electrical conductivity (–)
$\alpha_{nf}$: Thermal diffusivity of nanofluid
$\tau_{zx} ,\tau_{zy}$: Shear stress near *x-* and *y-* direction
$$\nu$$: Kinematic viscosity $$\left( {{\text{m}}^{2} {\text{s}}^{ - 1} } \right)$$
$\nu_{f}$: Kinematic viscosity of the fluid (–)
$\nu_{nf}$: Nanofluid kinematic viscosity (–)
$\tau_{0}$: Thermal relaxation characteristic time (> 0 )
$\gamma$: Thermal relaxation coefficient (dimensionless)
$\beta_{H}$: Hall parameter (dimensionless)
$\beta_{i}$: Ion slip parameter (dimensionless)
